# IMproving Preclinical Assessment of Cardioprotective Therapies (IMPACT): a small animal acute myocardial infarction randomized-controlled multicenter study on the effect of ischemic preconditioning

**DOI:** 10.1007/s00395-025-01102-3

**Published:** 2025-03-12

**Authors:** Sauri Hernandez-Resendiz, Reinis Vilskersts, David Aluja, Ioanna Andreadou, Péter Bencsik, Maija Dambrova, Panagiotis Efentakis, Fei Gao, Zoltán Giricz, Gábor B. Brenner, Nabil V. Sayour, Tamás G. Gergely, András Makkos, Javier Inserte, Roisin Kelly-Laubscher, Attila Kiss, Thomas Krieg, Brenda R. Kwak, Sandrine Lecour, Gary Lopaschuk, Michał Mączewski, Michał Waszkiewicz, Marta Oknińska, Pasquale Pagliaro, Bruno Podesser, Hiran A. Prag, Marisol Ruiz-Meana, Tamara Szabados, Coert J. Zuurbier, Péter Ferdinandy, Derek J. Hausenloy

**Affiliations:** 1 Cardiovascular and Metabolic Disorders Programme, Duke-NUS Medical School, 8 College Road, Singapore, Singapore; 2 National Heart Research Institute Singapore, National Heart Centre Singapore, Singapore, Singapore; 3 Laboratory of Pharmaceutical Pharmacology, Latvian Institute of Organic Synthesis, Riga, Latvia; 4 Department of Pharmaceutical Chemistry, Riga Stradins University, Riga, Latvia; 5 Vall d’hebron Institut de Recerca (VHIR), Hospital Universitari Vall d’Hebron, Barcelona, Spain; 6 Centro de Investigación Biomédica en Red Cardiovascular (CIBER-CV), Instituto de Salud Carlos III, Madrid, Spain; 7 Laboratory of Pharmacology, Faculty of Pharmacy, National and Kapodistrian University of Athens, Athens, Greece; 8 Department of Pharmacology and Pharmacotherapy, Albert-Szent-Györgyi Medical School, University of Szeged, Szeged, Hungary; 9 Pharmahungary Group, Szeged, Hungary; 10 Centre for Quantitative Medicine (CQM), Duke-NUS Medical School, Singapore, Singapore; 11 MTA-se System Pharmacology Research Group, Department of Pharmacology and Pharmacotherapy, Semmelweis University, Nagyvárad Tér 4, Budapest, 1089 Hungary; 12 Department of Pharmacology and Therapeutics, School of Medicine, College of Medicine and Health, University College Cork, Cork, Ireland; 13 Ludwig Boltzmann Institute for Cardiovascular Research at Center for Biomedical Research and Translational Surgery, Medical University of Vienna, Wien, Austria; 14 Department of Medicine, University of Cambridge, Cambridge, UK; 15 Department of Pathology and Immunology, and Geneva Center for Inflammation Research, Faculty of Medicine, University of Geneva, Geneva, Switzerland; 16 Cape Heart Institute, Faculty of Health Sciences, University of Cape Town, Cape Town, South Africa; 17 Cardiovascular Research Centre, University of Alberta, Edmonton, Canada; 18 Department of Clinical Physiology, Centre of Postgraduate Medical Education, Warsaw, Poland; 19 Clinical and Biological Science Department, University of Turin, Turin, Italy; 20 Amsterdam UMC, Location AMC, Department of Anaesthesiology, Laboratory of Experimental Intensive Care and Anaesthesiology (L.E.I.C.A.), Amsterdam Cardiovascular Sciences, Amsterdam, The Netherlands; 21 Yong Loo Lin School of Medicine, National University Singapore, Singapore, Singapore; 22 The Hatter Cardiovascular Institute, University College London, London, UK

**Keywords:** Ischemic preconditioning, Ischemia/reperfusion injury, Randomized controlled trial, Acute myocardial infarction, Multisite network, Small animal models

## Abstract

Although many cardioprotective interventions have been shown to limit infarct size (IS), in preclinical animal studies of acute myocardial ischemia/reperfusion injury (IRI), their clinical translation to patient benefit has been largely disappointing. A major factor is the lack of rigor and reproducibility in the preclinical studies. To address this, we have established the IMproving Preclinical Assessment of Cardioprotective Therapies (IMPACT) small animal multisite acute myocardial infarction (AMI) network, with centralized randomization and blinded core laboratory IS analysis, and have validated the network using ischemic preconditioning (IPC). Eight sites from the COST Innovators Grant (IG16225) network participated in the IMPACT AMI study. Mice and rats were randomly allocated into Sham, Control, or IPC groups. The IRI group underwent 45 min (mice) or 30 min (rats) of left coronary artery occlusion followed by 24 h reperfusion. IPC comprised three cycles of 5 min occlusion/reperfusion before IRI. IS was determined by a blinded core lab. The majority of site showed significant cardioprotection with IPC. In pooled mouse data, IPC (*N* = 42) reduced IS/AAR by 35% compared to control (*N* = 48) (30 ± 16% versus 46 ± 13%; *p* < 0.005), and in rat data, IPC (*N* = 36) reduced IS/AAR by 29% when compared to control (*N* = 39) (32 ± 19% versus 45 ± 14%; *p* < 0.01). The IMPACT multisite mouse and rat AMI networks, with centralized randomization and blinded core IS analysis, were established to improve the reproducibility of cardioprotective interventions in preclinical studies and to facilitate the translation of these therapies for patient benefit.

**Supplementary Information:**

The online version contains supplementary material available at 10.1007/s00395-025-01102-3.

## Introduction

Acute myocardial infarction (AMI) and the subsequent heart failure (HF) are among the leading causes of death and disability in Europe and worldwide. As such, new treatments are needed to reduce myocardial infarct size (IS) (termed ‘cardioprotection’), preserve cardiac function, and prevent the onset of HF. Although many therapies have been reported to reduce myocardial IS in preclinical acute myocardial ischemia/reperfusion injury (IRI) studies, their translation into the clinical setting for patient benefit has been disappointing [10, 14, 16, 18].

A primary obstacle to translating cardioprotective therapies into clinical practice has been the lack of systematic and robust preclinical evaluation of their efficacy before embarking on clinical testing. This includes publication bias against neutral studies and the failure to undertake prospective sample size estimations to reliably detect a true cardioprotective effect if one exists [25, 33]. Indeed, recent meta-analyses of preclinical studies showed large heterogeneity in study design and reporting quality [6, 31]. Moreover, one of these meta-analyses showed a discrepancy between the meta-analysis and a three-center in vivo study [31]. These results underscore the need for randomized-controlled multicenter preclinical studies in cardioprotection. The need for a multisite network to improve the rigor and reproducibility of preclinical cardioprotection studies was first tested in the National Heart, Lung, and Blood Institute (NHLBI)-funded Animal Models for Protecting Ischemic Myocardium (AMPIM) program [29]. Thirty years later, the Consortium for preclinicAl assESsment of cARdioprotective interventions (CAESAR) research network with only two sites performed AMI studies in mice, rabbits, and pigs [26]. Unfortunately, this initiative is no longer active, leaving a gap in the field for tools to bridge preclinical findings with clinical translation. More recently, the CIBERCV Cardioprotection Large Animal Platform (CIBER-CLAP) emerged in Spain to address experimental reproducibility before moving toward clinical application. However, this network has yet to publish any results [30]. The EU-CARDIOPROTECTION COST Action (CA16225), a large network of cardioprotection researchers, has published guidelines aimed at improving the reliability and rigor of preclinical cardioprotection studies [4] and have also introduced the IMproving Preclinical Assessment of Cardioprotective Therapies (IMPACT) criteria, a detailed step-by-step framework aimed at improving the reproducibility of preclinical cardioprotection studies [25]. Our recent multicenter study, conducted in pigs, successfully validated the effects of ischemic preconditioning (IPC) while underscoring the challenges associated with variability and translational gaps in preclinical research [23]. The IMPACT small animal network was designed as a complementary platform to bridge the gap between small and large animal models of AMI. By incorporating centralized randomization, blinded infarct size analysis, and rigorous multicenter collaboration, the small animal network provides a robust and validated framework for assessing cardioprotective therapies. This study highlights the outcomes of this novel small animal network, emphasizing its potential to enhance rigor, reproducibility, and translatability in preclinical research.

## Methods

### The IMPACT small animal network

The IMPACT small animal network was centrally coordinated by an IMPACT working group comprising: (1) the principal investigators and team members from each of the participating sites who performed the IRI studies; (2) the IMPACT Image Analysis Core Lab (IACL), which performed the centralized core laboratory IS analysis (Latvian Institute of Organic Synthesis, Riga, Latvia); (3) the IMPACT statistical core (Duke-NUS/NHCS, Singapore) who provided the central randomization lists to the sites and undertook all the statistical analyses; and (4) external advisors, who were not participants in the network but provided advice to the network.

Prior to commencing studies for the IMPACT network, each site underwent rigorous quality control assessment of their infarct images by the IMPACT IACL, with individual feedback provided to each site for optimization of the infarct images (Table S1). Eight different sites in the IG16225 network that agreed to participate in the IMPACT small animal multisite AMI network passed the quality control process and participated in the AMI network studies as follows:

Mice-Sites (M-S): M-Site 1—University of Szeged and Pharmahungary Group (Szeged, Hungary); M-Site 2—University of Cambridge (Cambridge, UK); M-Site 3—Vall’Hebron Institut de Recerca (VHIR) (Barcelona, Spain); M-Site 4—Duke-National University Singapore (Duke-NUS) Medical School and National Heart Centre Singapore (NHCS) (Singapore); and M-Site 5—National and Kapodistrian University of Athens (Athens, Greece).

Rats-Sites (R-S): R-Site 1—Semmelweis University and Pharmahungary Group (Budapest, Hungary); R-Site 2—Duke-NUS and NHCS (Singapore); R-Site 3—Latvian Institute of Organic Synthesis (Riga, Latvia); and R-Site 4—Centre of Postgraduate Medical Education (Warsaw, Poland).

Most of the participating sites had previously established mice and/or rat IRI models in their respective laboratories.

### Animals

All experimental protocols conformed to the Directive 2010/63/EU of the European Parliament on the protection of animals used for scientific purposes and the Animal Research: Reporting of In Vivo Experiments (ARRIVE) guidelines [28]. The experimental protocols were formally approved by the appropriate national or institutional ethics committees.

Male C57BL/6 J and C57BL/6N mice (aged 8–14 weeks, body weight ~ 23–30 g) were kept in specific pathogen-free conditions with unrestricted access to food and water according to local husbandry conditions at each site (Table S2A). Male Sprague–Dawley and Wistar rats, aged 8–12 weeks and weighing between ~ 230–300 g, were kept with unrestricted access to food and water according to local husbandry conditions at each site (Table S2B).

### IMPACT small animal AMI protocols

**Fig. 1:**
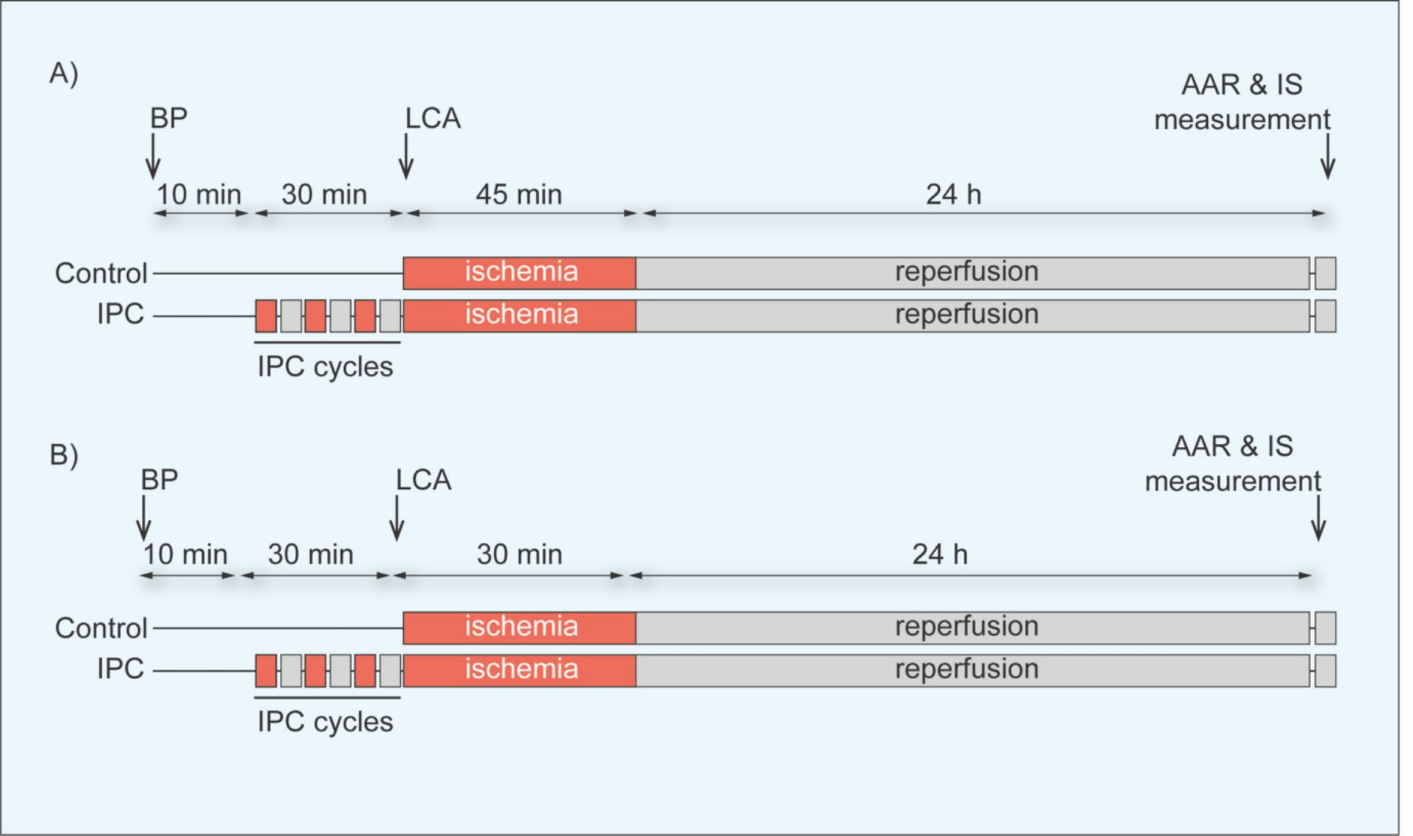
Overview of the experimental protocol used in the IMPACT small animal acute myocardial infarction networks. For both **A** mice and **B** rats, myocardial infarct size (IS) and the area-at-risk (AAR) were quantified using dual staining of triphenyl tetrazolium chloride (TTC) and Evans blue (EB). The staining process was conducted after 24 h reperfusion following IPC and occlusion of the left coronary artery (LCA). Ischemic preconditioning, IPC; left coronary artery, LCA; buprenorphine, BP; area-at-risk, AAR; infarct size, IS

The IMPACT working group drafted the study protocols in which both mice and rats were randomized to one of the following groups: (1) Sham, (2) Control, and (3) IPC groups. Pre-established centralized randomization lists generated by the IMPACT statistical core randomly assigned six animals to the sham group and 12 animals each to the control and IPC groups. These group sizes were determined based on prior experience at the test sites, published IPC studies, and expected mortality rates among animals subjected to acute IRI. In the Sham protocol, a minimally invasive thoracotomy was conducted, during which a ligature was placed around the left coronary artery (LCA) without inducing occlusion. In the control IRI protocol, mice underwent 45 min LCA ischemia and rats underwent 30 min of LCA ischemia, each followed by 24 h of reperfusion. In the IPC protocol, both mice and rats underwent three cycles of 5 min of LCA ischemia, interspersed with 5 min reperfusion, immediately before the index IRI, a protocol which is frequently used in published studies [38]. After 24 h of reperfusion, the IS and area-at-risk (AAR) ratio was assessed (Fig. 1).

The analgesic and anesthetic agents used for AMI surgery in mice and rats were as follows: *Mice: *Buprenorphine at 0.05–0.5 mg/kg administered subcutaneously as a single bolus 10 min before surgery for premedication. For anesthesia, ketamine was given at 50–100 mg/kg intraperitoneally as a single bolus 10 min before surgery, with M-Site 3 administering an additional bolus of ~ 16 mg/kg if needed. Xylazine was administered at 10–20 mg/kg intraperitoneally as a single bolus 10 min before surgery. M-Site 1 utilized isoflurane at 5% for induction and 1.5–2% for maintenance throughout the surgery. M-Sites 2 and 4 used 0.5% isoflurane during the surgery if needed. Detailed anesthetic protocols across the mice sites are summarized in Table S3A.

*Rat:* For premedication, buprenorphine was administered subcutaneously at a dose of 0.05 mg/kg as a single bolus, ensuring the safety and well-being of the animals. At R-Site 1, it was given 15 min before surgery. At R-Site 2, it was administered 10 min before surgery, and at R-Site 3, it was given 30 min before LCA ligation. For anesthesia, pentobarbital at 60 mg/kg, ketamine at 80–100 mg/kg, and xylazine at 10–20 mg/kg were administered intraperitoneally as a single bolus, with utmost care and attention to safety measures. Isoflurane was used for maintenance during the surgery at a concentration of 0.5–2% at R-Site 2 and R-Site 4. Detailed anesthetic protocols across the rat sites are summarized in Table S3B.

The methods used for euthanasia in mice and rats are variable between sites. For mice, the methods included intraperitoneal administration of pentobarbital at doses of 90 mg/kg or 100 mg/kg, or ketamine at 100 mg/kg combined with xylazine at 20 mg/kg. Additionally, some protocols involved the use of isoflurane at 5%. For rats, euthanasia methods included intraperitoneal administration of pentobarbital at 60 mg/kg or 100 mg/kg, or ketamine at 100 mg/kg combined with xylazine at 20 mg/kg, with some protocols also using heparin at 1000 IU/kg. These methods ensured humane treatment and were crucial for subsequent procedures. Detailed protocols for both mice and rats are summarized in Tables S5A and S5B, respectively.

Predefined exclusion criteria included: failure to induce ischemia with LCA ligation, failure of reperfusion following removal of occlusion, damage to the LCA during needle insertion or occlusion and reperfusion, ventilation problems during surgery, and uncontrolled bleeding. For details of the preoperative preparation procedure, surgical procedure, postsurgical care and analgesia, and measurement of IS at each of the sites, please see Tables S3–S5.

### Central measurement of infarct size by the IMPACT image analysis core lab

The IMPACT IACL analyzed the infarct images from all sites in a blinded fashion using computerized planimetry with Image-Pro Plus version 6.3 (Media Cybernetics). The laboratory measured IS as the non-TTC-stained area within the left ventricle (LV) and calculated the area-at-risk (AAR) as the total LV region unstained by Evans blue (EB) or methylene blue (MB). Additionally, non-AAR was determined by the EB-stained region and viable myocardium by the TTC-stained area within the AAR. The primary study outcome was the IS/AAR%. The exclusion criteria for image analysis included an IS/AAR% of ≥ 90% or < 10% for infarcted hearts or an AAR/LV ratio of < 10%. The exclusion criterion of hearts with an IS/AAR ratio < 10% was chosen due to the variability inherent in the coronary artery ligation model which is well documented for producing a board range of infarct sizes between 10 and 70%, even when suture is placed in the same anatomical location [7, 27]. The exclusion criterion of IS/AAR% < 10% was applied only to heart images from the Control and IPC groups.

### Central randomization and statistical analyses by the IMPACT statistics core

The IMPACT Statistics Core provided the central randomization list to each site, performed data analysis unblinding following completion of all IRI studies and undertook the statistical analysis for all the IRI studies. A sequence of treatments (Sham, Control, and IPC) randomly permuted was generated for each center and a central randomization list was provided to each site for 30 animals (*N* = 6 Sham, *N* = 12 Control, and *N* = 12 IPC) to allow for exclusions and mortality. Myocardial AAR, IS, IS/AAR, and mortality rates were analyzed separately. Data were tested for normal distribution using the Shapiro–Wilk test. The myocardial AAR, IS, and the ratio of IS/AAR were analyzed using the Wilcoxon rank-sum test and pooled data analysis using two-way ANOVA in STATA version 18. Mortality rates among treatments (Sham, Control, and IPC) are compared using Fisher’s exact test (STATA version 18).

## Results

### Study exclusions and mortality rates

Details of animal exclusions are listed in Tables S6A and S6B.

### Cardioprotective efficacy of IPC in IMPACT mice AMI network

**Fig. 2:**
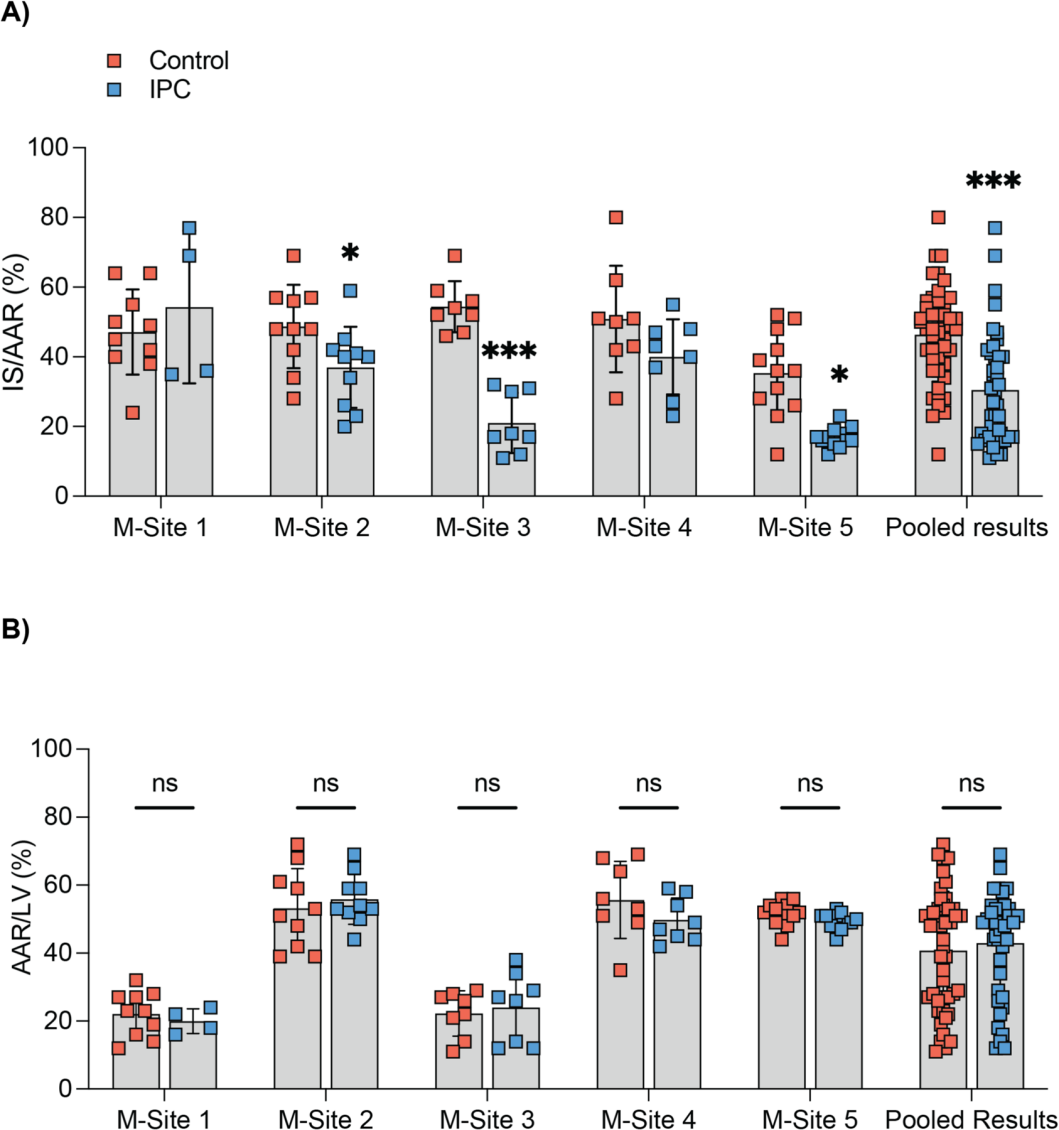
Site-specific analysis of area-at-risk and infarct size in mice hearts. **A** Site-specific analysis of mean infarct size/area-at-risk (IS/AAR%) showed IPC significantly reducing IS/AAR% when compared to control. **B** Site-specific analysis of mean area-at-risk/left ventricle volume (AAR/LV%) showed no significant difference between control and IPC at each site. However, there were significant differences in AAR/LV% in both control and IPC between the sites (*p* < 0.001 ANOVA). Results are presented as mean ± SD, with significance levels indicated; *p < 0.05, **p < 0.01 ***p < 0.005 vs. control group, Wilcoxon rank

In the pooled analysis of five sites, there were no significant differences in AAR/LV% between the Control and IPC groups (42 ± 17% vs. 43 ± 15%, respectively; *p* = ns; Fig. 2A). However, there were significant differences in AAR/LV% between sites (*p* < 0.001). In the pooled analysis, IPC significantly reduced IS/AAR% by 35% when compared to the control group (30 ± 16% vs. 46 ± 13%, respectively; *p* < 0.005; Fig. 2B). In evaluation of IS/AAR% from the individual sites, three sites (M-Sites 2, 3, and 5) showed significant cardioprotection with IPC [10], with one site (M-Site 4) showing a non-significant reduction in IS, and one site (M-Site 1) failing to show IS reduction with IPC (Fig. 2A). The IS/AAR% in the sham group was relatively high (pooled analysis of 5 sites 16 ± 13%) with variability across the sites ranging from 3 ± 2% in M-Site 5 to 25.0 ± 20.1% in M-Site 2 (Suppl. Figure 1).

### Cardioprotective efficacy of IPC in IMPACT rat AMI network

**Fig. 3:**
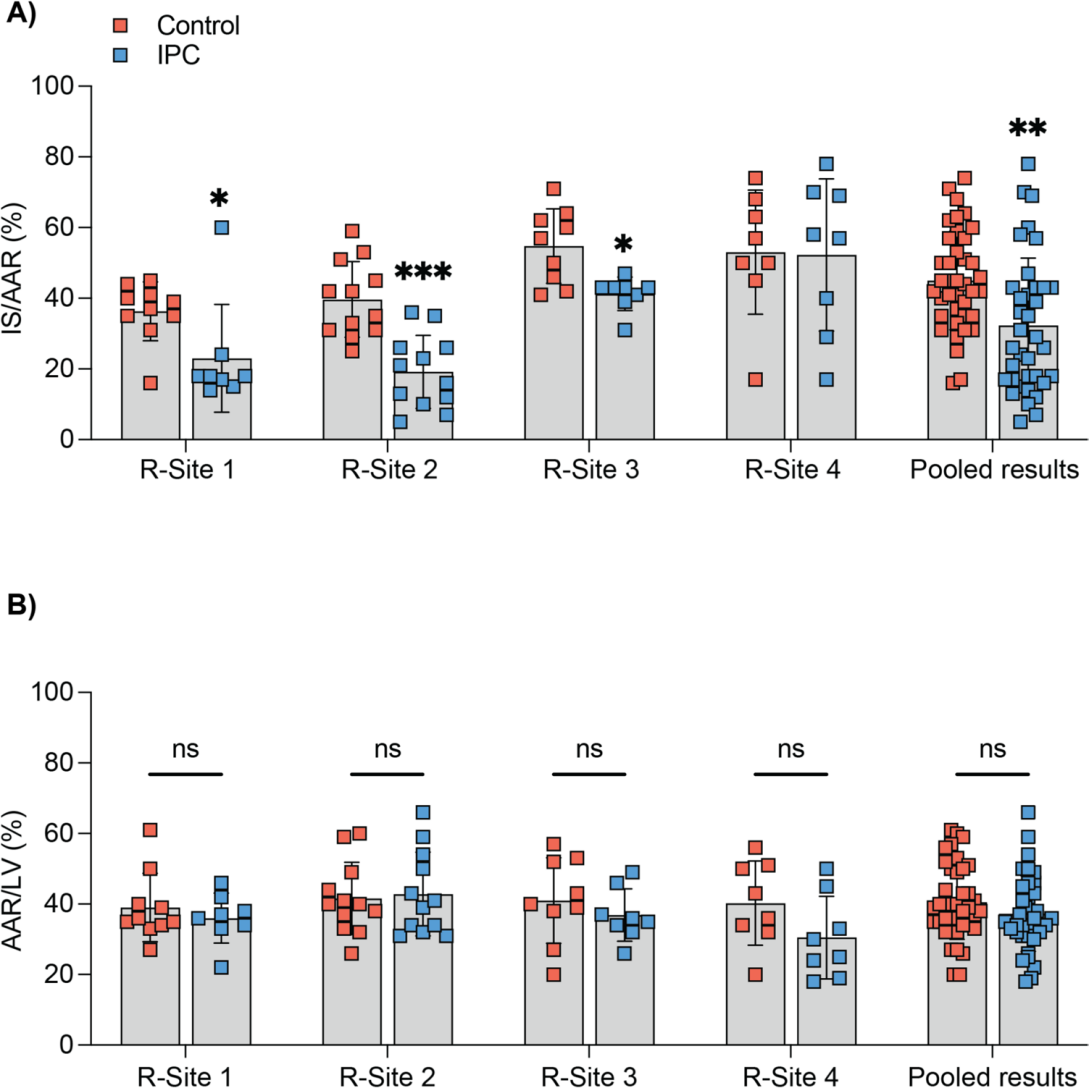
Site-specific analysis of area-at-risk and infarct size in rat hearts. **A** Site-specific analysis of mean infarct size/area-at-risk (IS/AAR%) showed IPC significantly reducing IS/AAR% when compared to control. **B** Site-specific analysis of mean area-at-risk/left ventricle volume (AAR/LV%) showed no significant differences between control and IPC at each site or between sites. Results are presented as mean ± SD, with significance levels indicated; *p<0.05, **p<0.01 ***p<0.005 vs. Control group, Wilcoxon rank

In the pooled analysis of four sites, there were no significant differences in AAR/LV% between Control and IPC groups (41 ± 11% vs. 39 ± 9%; *p* = ns; Fig. 3A). IPC significantly reduced IS/AAR% by 29% when compared to the control group in the pooled analysis of four sites (32 ± 19% vs. 45 ± 14%, respectively; *p* < 0.01; Fig. 3B). In evaluation of IS/AAR% from the individual sites, three sites (R-Sites 1–3) showed significant cardioprotection with IPC, with one site (R-Site 4) failing to show IS reduction with IPC (Fig. 3A). In R-Sites 1–3, the IS/AAR% in the sham group was lower than in the mice sites ranging from 6 ± 3% to 9 ± 7%, but it was very high in R-Site 4 (36 ± 27%) (Suppl. Figure 2).

### Surgical mortality rates

**Fig. 4:**
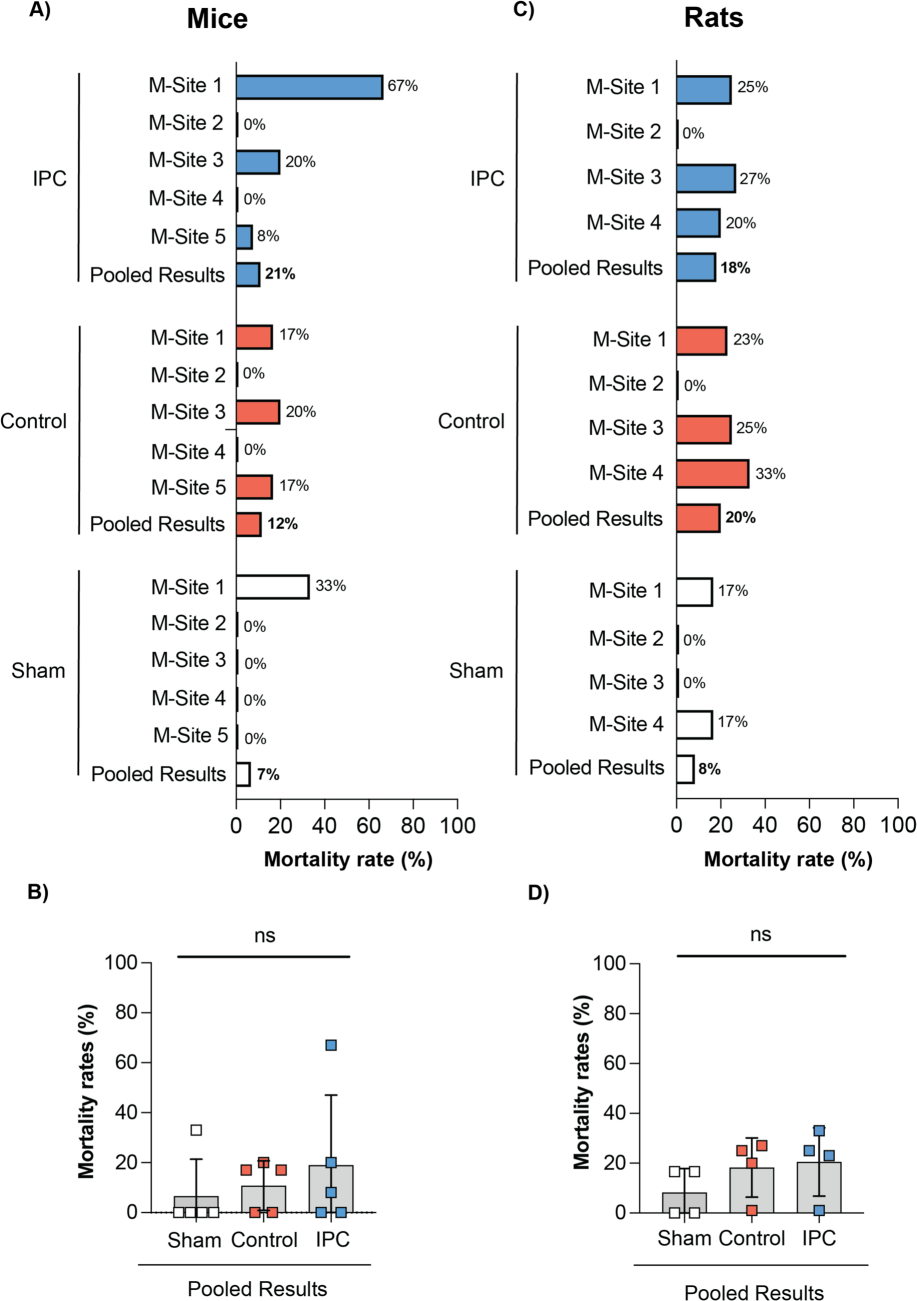
Mortality data in mouse and rat hearts. **A** Mortality rates for mice subjected to sham (white bars), control (red bars) and IPC (blue bars) at the 5 different sites. **B** Pooled mortality rates for mice subjected to sham (white squares), control (red squares) and IPC (blue squares), indicating no significant differences between the groups. **C** Mortality rates for rats subjected to sham (white bars), control (red bars) and IPC (blue bars) at the 4 different sites. **D** Pooled mortality rates for rats subjected to sham (white squares), control (red squares) and IPC (blue squares), indicating no significant differences between the groups

Details of surgical mortality are listed in Table S6B and displayed in Fig. 4. There was a high variation in mortality rates between the different sites ranging from 0 to 67% in the mouse sites and 0 to 33% in the rat sites. In M-sites 2 and 4 and R-Site 2, there were no mortalities in any of the groups, whereas in M-site 1, the mortality rate was 67% in the IPC group and, in R-Site 4, the mortality rate was 33%. Even in the sham groups, mortality rates of up to 33% and 17% were observed in the mouse and rat sites, respectively.

## Discussion

We have successfully established new mice and rat multisite AMI networks for evaluating the efficacy of novel cardioprotective therapies using centralized randomization and centralized blinded core lab analysis of IS, which should result in improved reproducibility of preclinical cardioprotection studies. We have demonstrated that the multisite approach for evaluating cardioprotective strategies is feasible. This was validated by demonstrating a reduction in IS using the established endogenous cardioprotection strategy of IPC [15, 19] across eight different sites when compared to control.

This study advances cardioprotection research with the application of a multisite randomized-controlled trial design (usually reserved for multicenter randomized clinical trials) to a preclinical animal AMI protocol, with: (1) centralized randomization and statistical analysis; (2) rigorous quality control in site selection based on optimization of IS images; (3) centralized and blinded core laboratory IS analysis; and (4) application of a standardized protocol across 8 different sites with pre-defined exclusion criteria. Variation in the cardioprotective efficacy of IPC between the sites would be expected and the aim of this study did not include investigating the reasons for these variations. According to the step-by-step IMPACT criteria for improving the preclinical assessment of promising cardioprotective interventions prior to their clinical evaluation, once the efficacy of the cardioprotective intervention has been demonstrated in a single-center, small animal IRI model, one should consider testing the intervention in a multicenter validation study in a minimum of three centers in at least one species (typically rat or mouse) [25]. The intention would be to validate study reproducibility using a network of research centers working in partnership to undertake small animal IRI studies to evaluate the cardioprotective intervention in a blinded fashion using standardized protocols and centralized core lab analysis of IS. In this regard, the IMPACT mice and rat AMI networks which have been established in this study would be suitable for this purpose. This is particularly important and will help address the issue of publication bias against neutral single-site cardioprotection studies [33].

The concept of a multicenter network of centers for undertaking in vivo preclinical evaluation of novel cardioprotective interventions was first tested in the National Heart, Lung, and Blood Institute (NHLBI)-funded Animal Models for Protecting Ischemic Myocardium (AMPIM) program of only three participating laboratories in open-chest dogs [29]. Thirty years later, the Consortium for preclinicAl assESsment of cARdioprotective interventions (CAESAR) research network with only two sites performed AMI studies in mice, rabbits, and pigs [1–3, 22]. Although the CAESAR consortium managed to demonstrate cardioprotection with IPC [22], it failed to reproduce cardioprotection with pharmacological agents which had been previously shown to be cardioprotective in single-site studies, such as nitrite and sildenafil [24]. These neutral studies were only published as abstracts highlighting the publication bias against neutral studies [33]. Due to lack of funding, the CAESAR consortium is no longer active, but it did succeed in demonstrating the utility of a multicenter network for evaluating the reproducibility of novel cardioprotective interventions.

Our IMPACT mouse and rat AMI networks established in the current study differ from the CAESAR consortium in several important aspects and introduce key advancements to address enhance the translatability of clinical cardioprotection. First, the IMPACT AMI network had a larger number of participating sites with eight sites undertaking mouse and/or rat AMI studies, compared to only three sites in the CAESAR consortium undertaking mouse, rabbit, and pig AMI experiments. Second, the CAESAR consortium adopted a strictly standardized approach, including uniform animal husbandry protocols, such as diet and housing conditions. While this method ensures controlled experimental settings, it often fails to capture the variability observed in clinical studies, where patient populations are inherently heterogeneous, and numerous factors influencing outcomes are not standardized. The IMPACT consortium, on the other hand, intentionally avoided standardizing various parameters, including diet, animal strain, anesthesia, analgesia, and day–night cycles. This approach more closely mimics the variability encountered in clinical trials, making the findings of the IMPACT consortium more representative of real-world conditions and enhancing their relevance and translatability to clinical settings. The effectiveness of novel cardioprotective treatments in preclinical research is influenced by a variety of factors including housing conditions, diet, sex, age, sedation, and anesthesia protocols. Within the framework of the IMPACT consortium, critical variables, such as sex, age, ischemia time, reperfusion time, and IPC protocol, were standardized to maintain consistency. Despite variations in rodent strains, housing, diet, and sedation/anesthetic regimens across the participating sites, our analysis indicates that these factors did not significantly affect study outcomes, underscoring the robustness of the cardioprotective responses to IPC observed in rodent models of AMI.

We included sham groups in our study, although the necessity for doing this in AMI studies has been debated in the literature. Evidence suggests that sham procedures do not significantly impact mortality, post-mortem findings, or physiological measures, and the sham procedure was only associated with modest alterations in inflammatory cytokine expression [21], suggesting that sham may not be required for cardioprotective efficacy studies. In our study, we observed relatively high IS/AAR% in sham mice (15–25%), and although the IS/AAR% ratios in 3 sham rat sites were lower (6–9%), R-Site 4 also had a very high IS/AAR% (36%) in the sham group. Moreover, we observed increased mortality in the sham groups at M-Site 1 and R-Site 4 which were unexpected findings. The cause of the high IS/AAR% in the sham groups is not clear but may relate to the surgical technique, e.g., damage to the LCA during the sham procedure. Similarly, the reasons for the unexpected mortality in the sham groups are not clear but may again be due to damage to the LCA during the sham procedure at R-Site 4 or due to the rib-cutting surgical procedure used at M-Site 1 for inducing AMI. This surgical approach is time-intensive, technically challenging, and associated with extensive tissue damage, heightened inflammation, and increased mortality rates, as previously reported [12, 34]. These results support the inclusion of sham groups in cardioprotection studies, as surgical procedure-induced myocardial injury and mortality may mask the cardioprotective or hidden cardiotoxic [5, 11] effects of a tested intervention.

The size of the AAR was found to vary considerably between the mouse sites being relatively small for M-Site 1 and M-Site 3, but not the rat sites—again, the reasons for this variation were not clear. Mortality rates also varied between sites with a very high mortality rate (67%) for the IPC group at M-Site 1 and no mortality at M-Site 2, M-Site 4, and R-Site 2. The reasons for the high mortality with IPC at M-Site 1 are not clear but may again be related to the rib-cutting technique used for inducing AMI. Two sites (M-Site 1 and R-Site 4) were unable to show cardioprotection with IPC. For M-Site 1, this may again relate to the rib-cutting technique used at this site and for R-Site 4, one possible explanation is the effect of reverse light–dark cycle at this site. It has been reported that the effect of preconditioning depends of the light–dark cycle in rats. The electrophysiological changes resulting from IPC are more effective mainly in the light (nonactive) part of the rat day regimen [35]. The reasons why M-Site 1 had one of the smaller AAR sizes, the highest mortality rates and failure to show cardioprotection with IPC are not clear and were not investigated in this study, but they may be due to the surgical technique, e.g., the rib-cutting technique used for inducing AMI.

These results illustrate the limitations in reproducibility of cardioprotection studies despite strict quality control implemented. The reason for this could not be identified by the available data, but differences in animal strains, size of AAR, the IRI protocol, mortality rate, the anesthesia or analgesia regimens, or individual non-responsiveness may explain the difference [20, 32]. The choice of anesthetic agents plays a critical role in cardioprotection studies, as different agents can influence experimental outcomes through their pharmacological effects. Volatile anaesthetics, such as isoflurane, confer myocardial protection against ischemia–reperfusion injury via preconditioning and postconditioning mechanisms [8, 37] which include reducing infarct size and improving contractile function recovery [8]. These cardioprotective effects are mediated by complex intracellular signaling pathways, such as the activation of ATP-sensitive potassium channels and the generation of reactive oxygen species [36]. However, for studying acute myocardial infarction in mice, we have decided to use the widely used anaesthetics ketamine and xylazine. However, we are aware that ketamine and xylazine can cause significant bradycardia and affect cardiac function, particularly at higher doses [39].

However, despite some sites not demonstrating cardioprotection with IPC, there was a significant reduction in IS with IPC in the pooled analyses, underscoring the high value of the multicenter network approach in this field. Although there is no positive control drug that can reproducibly reduce IS when administered at reperfusion, we believe that IPC can be used as a positive control intervention to demonstrate cardioprotection. However, the limitations of using IPC as the cardioprotective intervention include: (1) the actual mechanisms of IPC-induced protection are not known; (2) other non-IPC-related cardioprotective mechanisms exist; and (3) that the IPC intervention is applied prior to coronary artery occlusion and is not clinically relevant to cardioprotection in AMI patients.

The determination of potential confounding factors in the evaluation of cardioprotective interventions should be guided by the intervention mechanism as well as the resources and facilities at hand. It is advisable that researchers take into account variables, such as sex, age, prevalent metabolic conditions (e.g., diabetes, hypercholesterolemia), and comedications as recommended by recent guidelines and the IMPACT criteria for improving the reliability and reproducibility in the preclinical evaluation of novel cardioprotective therapies [10, 25]. According to the IMPACT criteria for improving the preclinical evaluation of novel cardioprotective therapies, the next step would be to test the infarct-limiting effects of the new treatment in a pig AMI model and if possible in a pig AMI network [10, 25]. In this regard, our IMPACT consortium has also established a new pig AMI network of 5 sites which has demonstrated cardioprotection with IPC and is now available for evaluating future novel cardioprotective therapies [23].

## Limitations

There are several limitations with our study. First, we observed large variations in IS within the sham and IPC groups across the eight sites. However, the primary aim of our study was to demonstrate that the small animal AMI networks could together demonstrate cardioprotection with the endogenous cardioprotective strategy of IPC and in this regard, we achieved our aim. The multicenter platform intentionally captured variability across sites, reflecting procedural, biological, and environmental differences that are frequently encountered in real-world preclinical research. Second, the study relied on Evans Blue and TTC staining as the primary endpoint for infarct size quantification. Although this is the gold standard, complementary endpoints such as coronary microvascular injury [16, 17], cardiac troponin levels or advanced imaging modalities like echocardiography [9] or MRI [13] could provide additional insights and should be considered in future studies. Third, the use of young, healthy male animals while providing a controlled environment for assessing IPC efficacy limits the clinical relevance of the findings [10]. To enhance translational applicability, we will incorporate models that better reflect clinical populations, including aged animals, animals with co-morbidities, and female animals in future studies. Finally, if we had collected information of the surgical mortality rates at M-Site 1 and R-Site 4 (the 2 sites which had higher than expected mortality rates), before preparing the central randomization lists, we could have taken this into consideration when deciding on the sample sizes per group for those sites. These limitations, while acknowledged, also provide opportunities for further development of the platform. Our study represents an important first step in establishing a multicenter small animal AMI network. However, additional refinements are necessary to realize its full potential. Future studies should incorporate hemodynamic monitoring, diverse animal models, and quality control measures to improve further the network's reproducibility and applicability to clinical research. These enhancements will strengthen the network's ability to address variability in cardioprotection studies and bridge the gap in clinical translation.

## Conclusion

We have established a new IMPACT multisite mouse and rat AMI network with centralized randomization and blinded core laboratory analysis of IS, demonstrating cardioprotection by IPC. Evaluating novel cardioprotective therapies in this network may help to increase the reproducibility and enhance clinical translation. While we acknowledge the limitations in the observed IPC variability, we believe that our multicenter approach represents a significant step forward in addressing the historical challenges of reproducibility in cardioprotection research. By including data from all sites and providing a detailed discussion of the factors influencing IPC efficacy, we aim to contribute to a more robust and clinically relevant preclinical research framework.

## Supplementary Information

Below is the link to the electronic supplementary material.

Supplementary file1 (DOCX 310 KB)

## Data Availability

The Supplementary Materials contain complete data availability and comprehensive tables of the significant resources. Additional datasets can be made available upon reasonable request.

## References

[1] Bolli R (2021) CAESAR’s legacy: a new era of rigor in preclinical studies of cardioprotection. Basic Res Cardiol 116:33. 10.1007/s00395-021-00874-8 34018051 10.1007/s00395-021-00874-8 PMC8137617

[2] Bolli RBL, Gross G, Mentzer R Jr, Balshaw D, Lathrop DA, NHLBI Working Group on the Translation of Therapies for Protecting the Heart from Ischemia (2004) Myocardial protection at a crossroads: the need for translation into clinical therapy. Circ Res 95:125–134. 10.1161/01.RES.0000137171.97172.d7 15271864 10.1161/01.RES.0000137171.97172.d7

[3] Bolli R, Tang XL (2022) New insights into cardioprotection, gained by adopting the CAESAR standards of rigor. Basic Res Cardiol 117:57. 10.1007/s00395-022-00964-1 36367590 10.1007/s00395-022-00964-1

[4] Botker HE, Hausenloy D, Andreadou I, Antonucci S, Boengler K, Davidson SM, Deshwal S, Devaux Y, Di Lisa F, Di Sante M, Efentakis P, Femmino S, Garcia-Dorado D, Giricz Z, Ibanez B, Iliodromitis E, Kaludercic N, Kleinbongard P, Neuhauser M, Ovize M, Pagliaro P, Rahbek-Schmidt M, Ruiz-Meana M, Schluter KD, Schulz R, Skyschally A, Wilder C, Yellon DM, Ferdinandy P, Heusch G (2018) Practical guidelines for rigor and reproducibility in preclinical and clinical studies on cardioprotection. Basic Res Cardiol 113:39. 10.1007/s00395-018-0696-8 30120595 10.1007/s00395-018-0696-8 PMC6105267

[5] Brenner GB, Makkos A, Nagy CT, Onodi Z, Sayour NV, Gergely TG, Kiss B, Gorbe A, Saghy E, Zadori ZS, Lazar B, Baranyai T, Varga RS, Husti Z, Varro A, Tothfalusi L, Schulz R, Baczko I, Giricz Z, Ferdinandy P (2020) Hidden cardiotoxicity of rofecoxib can be revealed in experimental models of ischemia/reperfusion. Cells 9:1–17. 10.3390/cells9030551 10.3390/cells9030551 PMC7140447 32111102

[6] Bromage DI, Pickard JM, Rossello X, Ziff OJ, Burke N, Yellon DM, Davidson SM (2017) Remote ischaemic conditioning reduces infarct size in animal in vivo models of ischaemia-reperfusion injury: a systematic review and meta-analysis. Cardiovasc Res 113:288–297. 10.1093/cvr/cvw219 28028069 10.1093/cvr/cvw219 PMC5408955

[7] Dawson D, Lygate CA, Saunders J, Schneider JE, Ye X, Hulbert K, Noble JA, Neubauer S (2004) Quantitative 3-dimensional echocardiography for accurate and rapid cardiac phenotype characterization in mice. Circulation 110:1632–1637. 10.1161/01.CIR.0000142049.14227.AD 15364813 10.1161/01.CIR.0000142049.14227.AD

[8] De Hert SG (2005) The concept of anaesthetic-induced cardioprotection: clinical relevance. Best Pract Res Clin Anaesthesiol 19:445–459. 10.1016/j.bpa.2005.02.004 16013693 10.1016/j.bpa.2005.02.004

[9] dos Santos L, Mello AF, Antonio EL, Tucci PJ (2008) Determination of myocardial infarction size in rats by echocardiography and tetrazolium staining: correlation, agreements, and simplifications. Braz J Med Biol Res 41:199–201. 10.1590/s0100-879x2008005000007 18246281 10.1590/s0100-879x2008005000007

[10] Ferdinandy P, Andreadou I, Baxter GF, Botker HE, Davidson SM, Dobrev D, Gersh BJ, Heusch G, Lecour S, Ruiz-Meana M, Zuurbier CJ, Hausenloy DJ, Schulz R (2023) Interaction of cardiovascular nonmodifiable risk factors, comorbidities and comedications with ischemia/reperfusion injury and cardioprotection by pharmacological treatments and ischemic conditioning. Pharmacol Rev 75:159–216. 10.1124/pharmrev.121.000348 36753049 10.1124/pharmrev.121.000348 PMC9832381

[11] Ferdinandy P, Baczko I, Bencsik P, Giricz Z, Gorbe A, Pacher P, Varga ZV, Varro A, Schulz R (2019) Definition of hidden drug cardiotoxicity: paradigm change in cardiac safety testing and its clinical implications. Eur Heart J 40:1771–1777. 10.1093/eurheartj/ehy365 29982507 10.1093/eurheartj/ehy365 PMC6554653

[12] Gao E, Lei YH, Shang X, Huang ZM, Zuo L, Boucher M, Fan Q, Chuprun JK, Ma XL, Koch WJ (2010) A novel and efficient model of coronary artery ligation and myocardial infarction in the mouse. Circ Res 107:1445–1453. 10.1161/CIRCRESAHA.110.223925 20966393 10.1161/CIRCRESAHA.110.223925 PMC3005817

[13] Grieve SM, Mazhar J, Callaghan F, Kok CY, Tandy S, Bhindi R, Figtree GA (2014) Automated quantification of myocardial salvage in a rat model of ischemia-reperfusion injury using 3D high-resolution magnetic resonance imaging (MRI). J Am Heart Assoc. 10.1161/JAHA.114.000956 25146703 10.1161/JAHA.114.000956 PMC4310382

[14] Hahn JY, Yu CW, Park HS, Song YB, Kim EK, Lee HJ, Bae JW, Chung WY, Choi SH, Choi JH, Bae JH, An KJ, Park JS, Oh JH, Kim SW, Hwang JY, Ryu JK, Lim DS, Gwon HC (2015) Long-term effects of ischemic postconditioning on clinical outcomes: 1-year follow-up of the POST randomized trial. Am Heart J 169:639–646. 10.1016/j.ahj.2015.01.015 25965711 10.1016/j.ahj.2015.01.015

[15] Hausenloy DJ, Barrabes JA, Botker HE, Davidson SM, Di Lisa F, Downey J, Engstrom T, Ferdinandy P, Carbrera-Fuentes HA, Heusch G, Ibanez B, Iliodromitis EK, Inserte J, Jennings R, Kalia N, Kharbanda R, Lecour S, Marber M, Miura T, Ovize M, Perez-Pinzon MA, Piper HM, Przyklenk K, Schmidt MR, Redington A, Ruiz-Meana M, Vilahur G, Vinten-Johansen J, Yellon DM, Garcia-Dorado D (2016) Ischaemic conditioning and targeting reperfusion injury: a 30 year voyage of discovery. Basic Res Cardiol 111:70. 10.1007/s00395-016-0588-8 27766474 10.1007/s00395-016-0588-8 PMC5073120

[16] Hausenloy DJ, Heusch G (2019) Translating cardioprotection for patient benefit: the EU-CARDIOPROTECTION COST action. J Am Coll Cardiol 73:2001–2003. 10.1016/j.jacc.2019.03.020 31000004 10.1016/j.jacc.2019.03.020

[17] Hernandez-Resendiz S, Palma-Flores C, De Los SS, Roman-Anguiano NG, Flores M, de la Pena A, Flores PL, Fernandez GJ, Coral-Vazquez RM, Zazueta C (2015) Reduction of no-reflow and reperfusion injury with the synthetic 17beta-aminoestrogen compound Prolame is associated with PI3K/Akt/eNOS signaling cascade. Basic Res Cardiol 110:1. 10.1007/s00395-015-0464-y 25589055 10.1007/s00395-015-0464-y

[18] Heusch G (2017) Critical issues for the translation of cardioprotection. Circ Res 120:1477–1486. 10.1161/CIRCRESAHA.117.310820 28450365 10.1161/CIRCRESAHA.117.310820

[19] Heusch G (2020) Myocardial ischaemia-reperfusion injury and cardioprotection in perspective. Nat Rev Cardiol 17:773–789. 10.1038/s41569-020-0403-y 32620851 10.1038/s41569-020-0403-y

[20] Heusch G, Botker HE, Ferdinandy P, Schulz R (2023) Primordial non-responsiveness: a neglected obstacle to cardioprotection. Eur Heart J 44:1687–1689. 10.1093/eurheartj/ehad160 36943315 10.1093/eurheartj/ehad160

[21] Iyer RP, de Castro Bras LE, Cannon PL, Ma Y, DeLeon-Pennell KY, Jung M, Flynn ER, Henry JB, Bratton DR, White JA, Fulton LK, Grady AW, Lindsey ML (2016) Defining the sham environment for post-myocardial infarction studies in mice. Am J Physiol Heart Circ Physiol 311:H822-836. 10.1152/ajpheart.00067.2016 27521418 10.1152/ajpheart.00067.2016 PMC5142180

[22] Jones SP, Tang XL, Guo Y, Steenbergen C, Lefer DJ, Kukreja RC, Kong M, Li Q, Bhushan S, Zhu X, Du J, Nong Y, Stowers HL, Kondo K, Hunt GN, Goodchild TT, Orr A, Chang CC, Ockaili R, Salloum FN, Bolli R (2015) The NHLBI-sponsored consortium for preclinicAl assESsment of cARdioprotective therapies (CAESAR): a new paradigm for rigorous, accurate, and reproducible evaluation of putative infarct-sparing interventions in mice, rabbits, and pigs. Circ Res 116:572–586. 10.1161/CIRCRESAHA.116.305462 25499773 10.1161/CIRCRESAHA.116.305462 PMC4329104

[23] Kleinbongard P, Arriola CG, Badimon L, Crisostomo V, Giricz Z, Gyongyosi M, Heusch G, Ibanez B, Kiss A, de Kleijn DPV, Podesser BK, Carracedo RR, Rodriguez-Sinovas A, Ruiz-Meana M, Sanchez Margallo FM, Vilahur G, Zamorano JL, Zaragoza C, Ferdinandy P, Hausenloy DJ (2024) The IMproving preclinical assessment of cardioprotective therapies (IMPACT): multicenter pig study on the effect of ischemic preconditioning. Basic Res Cardiol 119:893–909. 10.1007/s00395-024-01083-9 39422732 10.1007/s00395-024-01083-9 PMC11628588

[24] Kukreja R, Tang X-L, Lefer D, Steenbergen C, Jones S, Guo Y, Li Q, Kong M, Stowers H, Hunt G, Tokita Y, Wu W, Ockaili R, Salloum F, Book M, Du J, Bhushan S, Goodchild T, Chang C, Bolli R (2014) Administration of sildenafil at reperfusion fails to reduce infarct size: results from the CAESAR cardioprotection consortium. FASEB J 28:650

[25] Lecour S, Andreadou I, Botker HE, Davidson SM, Heusch G, Ruiz-Meana M, Schulz R, Zuurbier CJ, Ferdinandy P, Hausenloy DJ, On behalf of the European Union CCAC (2021) IMproving preclinical assessment of cardioprotective therapies (IMPACT) criteria: guidelines of the EU-CARDIOPROTECTION COST Action. Basic Res Cardiol 116:52. 10.1007/s00395-021-00893-5 34515837 10.1007/s00395-021-00893-5 PMC8437922

[26] Lefer DJ, Bolli R (2011) Development of an NIH consortium for preclinicAl AssESsment of CARdioprotective therapies (CAESAR): a paradigm shift in studies of infarct size limitation. J Cardiovasc Pharmacol Ther 16:332–339. 10.1177/1074248411414155 21821536 10.1177/1074248411414155

[27] Patten RD, Aronovitz MJ, Deras-Mejia L, Pandian NG, Hanak GG, Smith JJ, Mendelsohn ME, Konstam MA (1998) Ventricular remodeling in a mouse model of myocardial infarction. Am J Physiol 274:H1812-1820. 10.1152/ajpheart.1998.274.5.H1812 9612394 10.1152/ajpheart.1998.274.5.H1812

[28] Percie du Sert N, Hurst V, Ahluwalia A, Alam S, Avey MT, Baker M, Browne WJ, Clark A, Cuthill IC, Dirnagl U, Emerson M, Garner P, Holgate ST, Howells DW, Karp NA, Lazic SE, Lidster K, MacCallum CJ, Macleod M, Pearl EJ, Petersen OH, Rawle F, Reynolds P, Rooney K, Sena ES, Silberberg SD, Steckler T, Wurbel H (2020) The ARRIVE guidelines 2.0: Updated guidelines for reporting animal research. Br J Pharmacol 177:3617–3624. 10.1111/bph.15193 32662519 10.1111/bph.15193 PMC7393194

[29] Reimer KA, Jennings RB, Cobb FR, Murdock RH, Greenfield JC Jr, Becker LC, Bulkley BH, Hutchins GM, Schwartz RP Jr, Bailey KR et al (1985) Animal models for protecting ischemic myocardium: results of the NHLBI cooperative study. Comparison of unconscious and conscious dog models. Circ Res 56:651–665. 10.1161/01.res.56.5.651 3838923 10.1161/01.res.56.5.651

[30] Rossello X, Rodriguez-Sinovas A, Vilahur G, Crisostomo V, Jorge I, Zaragoza C, Zamorano JL, Bermejo J, Ordonez A, Bosca L, Vazquez J, Badimon L, Sanchez-Margallo FM, Fernandez-Aviles F, Garcia-Dorado D, Ibanez B (2019) CIBER-CLAP (CIBERCV cardioprotection large animal platform): a multicenter preclinical network for testing reproducibility in cardiovascular interventions. Sci Rep 9:20290. 10.1038/s41598-019-56613-6 31889088 10.1038/s41598-019-56613-6 PMC6937304

[31] Sayour NV, Brenner GB, Makkos A, Kiss B, Kovacshazi C, Gergely TG, Aukrust SG, Tian H, Zenkl V, Gomori K, Szabados T, Bencsik P, Heinen A, Schulz R, Baxter GF, Zuurbier CJ, Voko Z, Ferdinandy P, Giricz Z (2023) Cardioprotective efficacy of limb remote ischaemic preconditioning in rats: discrepancy between a meta-analysis and a three-centre in vivo study. Cardiovasc Res 119:1336–1351. 10.1093/cvr/cvad024 36718529 10.1093/cvr/cvad024 PMC10262179

[32] Schreckenberg R, Klein J, Kutsche HS, Schulz R, Gomori K, Bencsik P, Benczik B, Agg B, Saghy E, Ferdinandy P, Schluter KD (2020) Ischaemic post-conditioning in rats: responder and non-responder differ in transcriptome of mitochondrial proteins. J Cell Mol Med 24:5528–5541. 10.1111/jcmm.15209 32297702 10.1111/jcmm.15209 PMC7214154

[33] Skyschally A, Kleinbongard P, Neuhauser M, Heusch G (2024) “Expression of concern”: publication bias for positive preclinical cardioprotection studies. Basic Res Cardiol 119:397–402. 10.1007/s00395-024-01050-4

[34] Sun Q, Wang KK, Pan M, Zhou JP, Qiu XT, Wang ZY, Yang Z, Chen Y, Shen H, Gu QL, Fang LH, Zhang GG, Bai YP (2018) A minimally invasive approach to induce myocardial infarction in mice without thoracotomy. J Cell Mol Med 22:5208–5219. 10.1111/jcmm.13708 30589494 10.1111/jcmm.13708 PMC6201221

[35] Svorc P, Benacka R (2008) The effect of hypoxic myocardial preconditioning is highly dependent on the light-dark cycle in Wistar rats. Exp Clin Cardiol 13:204–208 19343168 PMC2663486

[36] Tanaka K, Ludwig LM, Kersten JR, Pagel PS, Warltier DC (2004) Mechanisms of cardioprotection by volatile anesthetics. Anesthesiology 100:707–721. 10.1097/00000542-200403000-00035 15108989 10.1097/00000542-200403000-00035

[37] Weber NC, Preckel B, Schlack W (2005) The effect of anaesthetics on the myocardium–new insights into myocardial protection. Eur J Anaesthesiol 22:647–657. 10.1017/s0265021505001080 16163910 10.1017/s0265021505001080

[38] Wever KE, Hooijmans CR, Riksen NP, Sterenborg TB, Sena ES, Ritskes-Hoitinga M, Warle MC (2015) Determinants of the efficacy of cardiac ischemic preconditioning: a systematic review and meta-analysis of animal studies. PLoS ONE 10:e0142021. 10.1371/journal.pone.0142021 26580958 10.1371/journal.pone.0142021 PMC4651366

[39] Xu Q, Ming Z, Dart AM, Du XJ (2007) Optimizing dosage of ketamine and xylazine in murine echocardiography. Clin Exp Pharmacol Physiol 34:499–507. 10.1111/j.1440-1681.2007.04601.x 17439422 10.1111/j.1440-1681.2007.04601.x

