# Correction: IMproving Preclinical Assessment of Cardioprotective Therapies (IMPACT): a small animal acute myocardial infarction randomized-controlled multicenter study on the effect of ischemic preconditioning

**DOI:** 10.1007/s00395-025-01142-9

**Published:** 2025-11-10

**Authors:** Sauri Hernandez-Resendiz, Reinis Vilskersts, David Aluja, Ioanna Andreadou, Péter Bencsik, Maija Dambrova, Panagiotis Efentakis, Fei Gao, Zoltán Giricz, Gábor B. Brenner, Nabil V. Sayour, Tamás G. Gergely, András Makkos, Javier Inserte, Roisin Kelly-Laubscher, Attila Kiss, Thomas Krieg, Brenda R. Kwak, Sandrine Lecour, Gary Lopaschuk, Michał Mączewski, Michał Waszkiewicz, Marta Oknińska, Pasquale Pagliaro, Bruno Podesser, Hiran A. Prag, Marisol Ruiz-Meana, Tamara Szabados, Coert J. Zuurbier, Péter Ferdinandy, Derek J. Hausenloy

**Affiliations:** 1 Cardiovascular and Metabolic Disorders Programme, Duke-NUS Medical School, 8 College Road, Singapore, Singapore; 2 National Heart Research Institute Singapore, National Heart Centre Singapore, Singapore, Singapore; 3 Laboratory of Pharmaceutical Pharmacology, Latvian Institute of Organic Synthesis, Riga, Latvia; 4 Department of Pharmaceutical Chemistry, Riga Stradins University, Riga, Latvia; 5 Vall d’hebron Institut de Recerca (VHIR), Hospital Universitari Vall d’Hebron, Barcelona, Spain; 6 Centro de Investigación Biomédica en Red Cardiovascular (CIBER-CV), Instituto de Salud Carlos III, Madrid, Spain; 7 Laboratory of Pharmacology, Faculty of Pharmacy, National and Kapodistrian University of Athens, Athens, Greece; 8 Department of Pharmacology and Pharmacotherapy, Albert-Szent-Györgyi Medical School, University of Szeged, Szeged, Hungary; 9 Present Address: Pharmahungary Group, Szeged, Hungary; 10 Centre for Quantitative Medicine (CQM), Duke-NUS Medical School, Singapore, Singapore; 11 MTA‑se System Pharmacology Research Group, Department of Pharmacology and Pharmacotherapy, Semmelweis University, Nagyvárad Tér 4, Budapest, 1089 Hungary; 12 Department of Pharmacology and Therapeutics, School of Medicine, College of Medicine and Health, University College Cork, Cork, Ireland; 13 Ludwig Boltzmann Institute for Cardiovascular Research at Center for Biomedical Research and Translational Surgery, Medical University of Vienna, Vienna, Austria; 14 Department of Medicine, University of Cambridge, Cambridge, UK; 15 Department of Pathology and Immunology, and Geneva Center for Inflammation Research, Faculty of Medicine, University of Geneva, Geneva, Switzerland; 16 Cape Heart Institute, Faculty of Health Sciences, University of Cape Town, Cape Town, South Africa; 17 Cardiovascular Research Centre, University of Alberta, Edmonton, Canada; 18 Department of Clinical Physiology, Centre of Postgraduate Medical Education, Warsaw, Poland; 19 Clinical and Biological Science Department, University of Turin, Turin, Italy; 20 Amsterdam UMC, Location AMC, Department of Anaesthesiology, Laboratory of Experimental Intensive Care and Anaesthesiology (L.E.I.C.A.), Amsterdam Cardiovascular Sciences, Amsterdam, The Netherlands; 21 Yong Loo Lin School of Medicine, National University Singapore, Singapore, Singapore; 22 The Hatter Cardiovascular Institute, University College London, London, UK

**Correction: Basic Research in Cardiology (2025) 120:335–346** 10.1007/s00395-025-01102-3

In the original article co-authors Gábor B. Brenner, András Makkos, Tamás G. Gergely and Nabil V. Sayour were missing from the author group due to an administrative error on the authors’ part.

The authors contributed significantly to the study by generating all data from R-site 1 (see Fig. 3 and Supplementary Fig. 2).

