# Supplementary material for: IMproving Preclinical Assessment of Cardioprotective Therapies (IMPACT): a small animal acute myocardial infarction randomized-controlled multicenter study on the effect of ischemic preconditioning
Source: Basic Res Cardiol. 2025 Mar 12;120(2):335–46. doi: 10.1007/s00395-025-01102-3 (PMC11976871; doi:10.1007/s00395-025-01102-3)
Supplement: Supplementary file 1 — Supplementary file1 (DOCX 310 KB) [file 395_2025_1102_MOESM1_ESM.docx]

***Supplementary Figures***

**
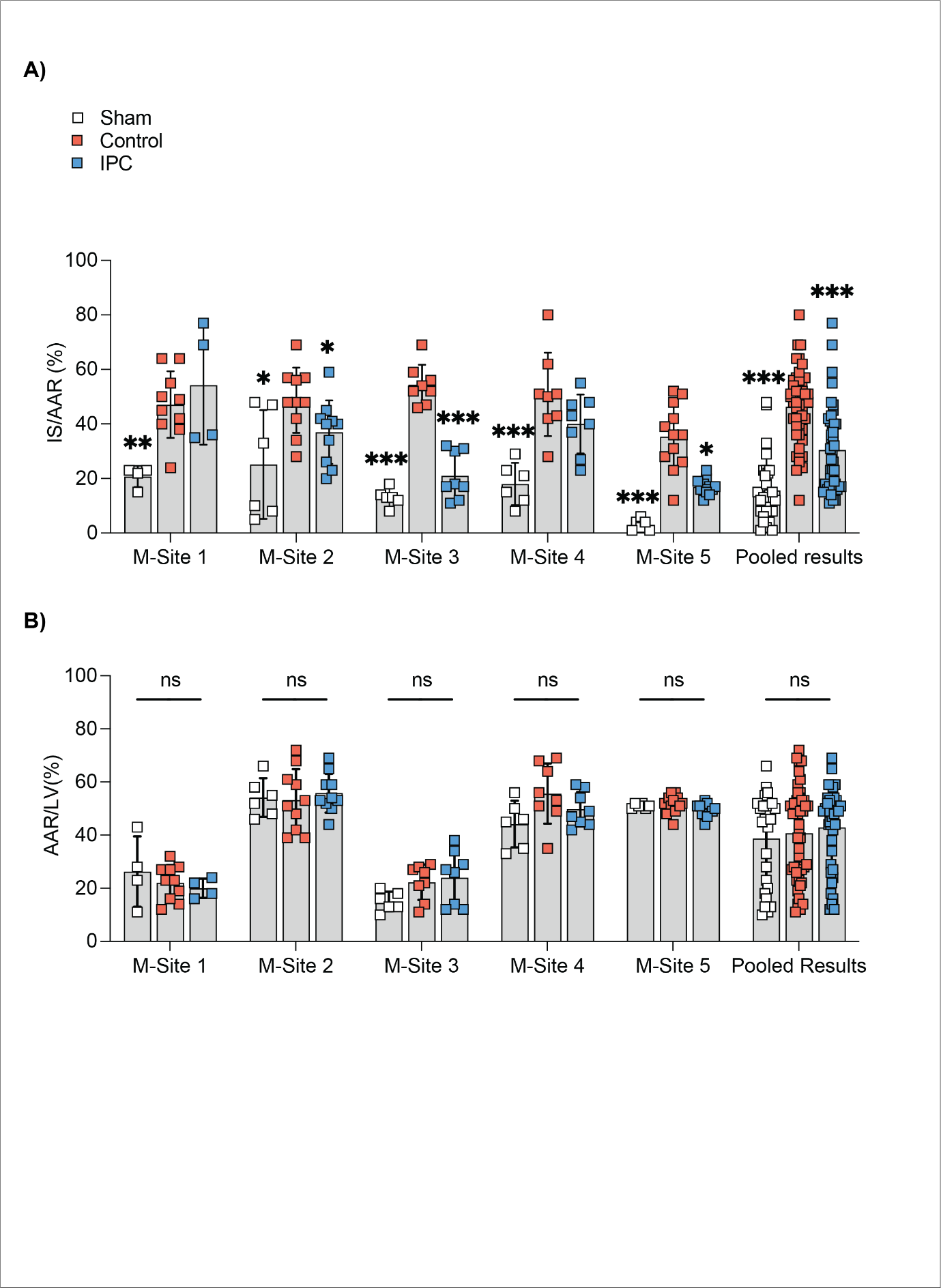
**

**Supplementary Figure 1.** Site-specific analysis of area-at-risk and infarct size in mice hearts

**(A)** Site-specific analysis of mean infarct size/area-at-risk (IS/AAR%) showed IPC significantly reducing IS/AAR% when compared to control.

**(B)** Site-specific analysis of mean area-at-risk/left ventricle volume (AAR/LV%) showed no significant difference between sham, control and IPC at each site. However, there were significant differences in AAR/LV% in both sham, control and IPC between the sites (p<0.001 ANOVA).

Results are presented as mean ± SD, with significance levels indicated; *p<0.05, **p<0.01 ***p<0.005 vs. Control group, Wilcoxon rank.


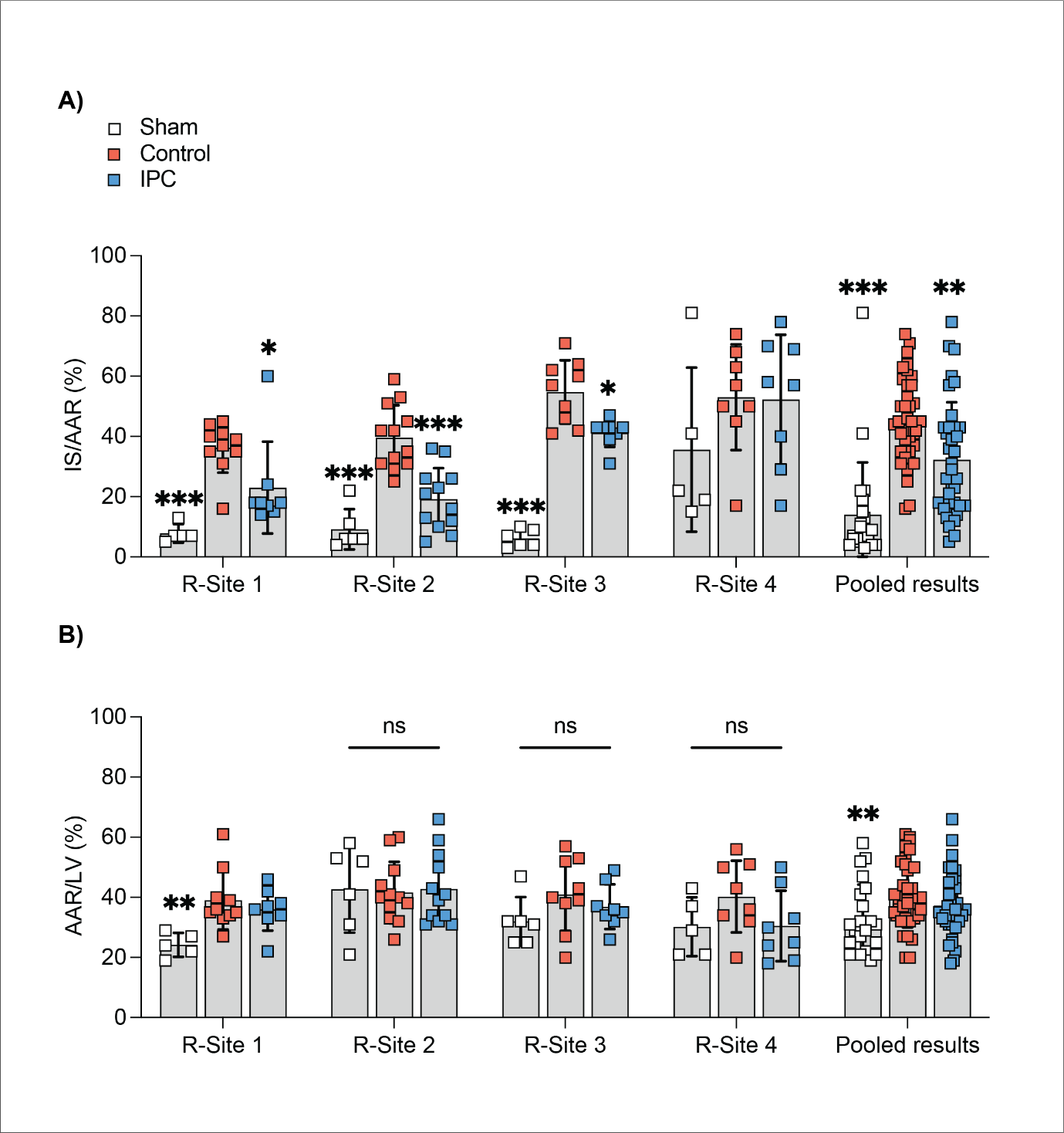


**Supplementary Figure 2.** Site-specific analysis of area-at-risk and infarct size in rat hearts

**(A)** Site-specific analysis of mean infarct size/area-at-risk (IS/AAR%) showed IPC significantly reducing IS/AAR% when compared to control.

**(B)** Site-specific analysis of mean area-at-risk/left ventricle volume (AAR/LV%) showed no significant differences between sham, control and IPC at each site or between sites.

Results are presented as mean ± SD, with significance levels indicated; *p<0.05, **p<0.01 ***p<0.005 vs. Control group, Wilcoxon rank.

***Supplementary Tables***

**Table S1.** Quality control (QC) criteria for the assessment of infarct size.

| **QC criteria** | **Variable** | **Exclusion criteria** | |
| --- | --- | --- | --- |
| **Tissue staining** | Achieve a clear and uniform delineation of areas of interest in heart slices for accurate and reliable quantification. | | Heart slices with unclear stained regions or not aligning with anatomical areas (e.g. presence of infarction in the right ventricle). |
| **Sectioning** | Slices uniformly sectioned (1-2 mm) to ensure even staining and clear visualization. | | Slices thicker or thinner than the specified range. |
| **TTC staining** | Performed TTC reaction (for 10min, at 37°C) to identify infarcted areas, ensuring consistent and clear delineation of viable and non-viable tissue. | | Inconsistent staining or unclear delineation between viable and non-viable tissue. |
| **Immersion in buffer** | Heart slices should be immersed in a buffer solution to prevent drying and preserve staining integrity. Heart slices should be immediately photographed. | | Heart slices showing signs of drying or discoloration. |
| **Uniform illumination** | Ensure heart slices are adequately illuminated with no prominent shadows for accurate imaging. | | Images with shadows or uneven illumination. |
| **Imaging** | High-resolution images should be captured of the slices under standardized lighting conditions.  Heart slices should be completely immersed in buffer solution to avoid reflections from the light source.  The images of the heart slices should be taken from the back and front. | | Images with poor resolution, poor lighting conditions, reflections affecting image clarity and missing views from either the back or front. |

**Table S2A.** Comparative overview of housing conditions and dietary protocols across mice sites.

|  | **Mouse weight and age during the experiment** | **Mouse strain** | **Breeder** | **Chow,**  **Producer** | **Contain soya** | **Reversed light-dark cycle** |
| --- | --- | --- | --- | --- | --- | --- |
| M-Site 1 | 23-29 g, 11-12-week-old | C57Bl/6N | Charles - River | SAFE® 132, OSAFE | Y | N |
| M-Site 2 | 25-30 g, 12-14-week-old | C57Bl/6J | Charles - River | R105-25, SAFE® | N | N |
| M-Site 3 | 25-30 g, 12-14-week-old | C57Bl/6N | Janvier Labs | SAFE® 150, OSAFE | N | N |
| M-Site 4 | 24-28 g, 8-10-week-old | C57Bl/6J | InVivos | Irradiated Mouse diet (SF00-100) | Y | N |
| M-Site 5 | ~25 g, 12-14-week-old | C57Bl/6J | Inhouse Breeding | Mucedola 4RF22, Mucedola | Y | N |

**Table S2B.** Comparative overview of housing conditions and dietary protocols across rat sites.

|  | **Rat weight and age during the experiment** | **Rat**  **strain** | **Breeder** | **Chow,**  **Producer** | **Contain soya** | **Reversed light-dark cycle** |
| --- | --- | --- | --- | --- | --- | --- |
| R-Site 1 | 238-295 g, 8-10-week old | Wistar | Toxicoop | Rat/Mouse Maintenance chow Sniff | Y | N |
| R-Site 2 | 250 -300 g, 8-10-week old | Sprague-Dawley | InVivos | Irradiated Rat diet (SF00-100) | Y | N |
| R-Site 3 | 250-300 g, 8-14-week old | Wistar | Envigo | R70 diet Lantmännen | N | N |
| R-Site 4 | 240-280 g, 8-10-week old | Wistar | Medical University of Bialystok | Standard diet | N | Y |

**Table S3A.** Anesthetic protocols and ventilation parameters across mice sites.

|  | **Premedication** | **Anesthesia** | **Ventilation parameters** | **Used gas** |
| --- | --- | --- | --- | --- |
| M-Site 1 | Buprenorphine 0.05 mg/Kg SC, 10 min before surgery | Isoflurane, 5% induction  1.5-2% maintenance | Volume controlled  RR, 120-150 strokes/min TV, 100-240 µl | 100% oxygen |
| M-Site 2 | Buprenorphine 0.05 mg/Kg SC, 10 min before surgery | Ketamine, 100 mg/Kg, IP  Xylazine, 10 mg/Kg, IP  Isoflurane | Volume controlled  RR, 120-150 breath/min  TV, 100-240 µl | 100% oxygen |
| M-Site 3 | None | Pentobarbital, 40 mg/Kg, IP  Ketamine, 50mg/Kg IP  1/3 of the initial dose if needed. | Volume controlled  RR, 133 breath/min  TV, 180 µl  PEEP 3 mbar | 100% oxygen |
| M-Site 4 | Buprenorphine 0.5 mg/Kg SC 10 min before surgery | Ketamine, 65 mg/Kg, IP  Xylazine, 13 mg/Kg, IP  Isoflurane, 0.5% | RR, 140 breath/min  TV, 160 µl  1% sigh  Breath 20 cmH_2_O | Air |
| M-Site 5 | Buprenorphine 0.075 mg/Kg SC, 10 min before surgery | Ketamine, 65 mg/Kg, IP  Xylazine, 20 mg/Kg, IP | Volume controlled  RR, 200 breath/min  TV, 250 µl | Air |

Subcutaneous, SC; intraperitoneal, IP; respiratory rate, RR; tidal volume, TV.

**Table S3B.** Anesthetic protocols and ventilation parameters across rat sites.

|  | **Premedication** | **Anesthesia** | **Ventilation parameters** | **Used gas** |
| --- | --- | --- | --- | --- |
| R-Site 1 | Buprenorphine 0.05 mg/Kg SC, 15 min before surgery | Pentobarbital, 60 mg/Kg IP | Volume controlled  RR, 74-78 strokes/min  TV, 1.47-1.78 ml | Air |
| R-Site 2 | Buprenorphine 0.05 – 0.1 mg/Kg SC, 10 min before surgery | Ketamine, 80-100 mg/Kg, IP  Xylazine, 10-20 mg/Kg, IP  Isoflurane, 0.5 - 2% | Volume controlled  RR, 60 breath/min  TV, 3 ml  1% sigh  Breath 20 cmH_2_O | Air |
| R-Site 3 | Atropine, 0.05 mg/Kg, Benzylpenicillin,150 mg/Kg, Buprenorphine, 0.05 mg/Kg SC,  30 min prior to LAD ligation | Ketamine, 100 mg/Kg, IP  Xylazine, 10mg/Kg, IP  Ketamine, 50 mg/Kg, IP (extra if needed) | Volume controlled  RR, 55 strokes/min  TV, 1.5 ml/100 g animal | Air |
| R-Site 4 | None | Ketamine, 100 mg/Kg, IP  Xylazine, 10 mg/Kg, IP  Isoflurane, 0.5% | Volume controlled  RR, 65 strokes/min  TV, 4.5-5.0 ml | Air enriched with oxygen |

Subcutaneous, SC; intraperitoneal, IP; respiratory rate, RR; tidal volume, TV.

**Table S4A.** Comparative overview of surgical techniques across mice sites.

|  | **Surgery** | **Parameters monitored during surgery** | **Temperature**  **range** | **Suture type for occlusion** | | **Analgesia** | |
| --- | --- | --- | --- | --- | --- | --- | --- |
| M-Site 1 | Thoracotomy by cutting one rib and intercostal muscles | ECG, body temperature | 36.5-37.5 °C | | 8-0 Prolene  (polypropylene) | | Buprenorphine 0.05 mg/Kg SC |
| M-Site 2 | Thoracotomy involving the dissection of intercostal muscles | ECG, PiP/PEEP, body temperature | 36.8-37.5 °C | | 7-0 prolene, 6.5mm needle, 1/2 circle | | Buprenorphine 0.05 mg/Kg SC |
| M-Site 3 | Thoracotomy involving the dissection of intercostal muscles | ECG, body temperature | 36.8-37.5 °C | | 6-0 braided, 9.3mm needle, 3/8 circle | | Buprenorphine 0.05 mg/Kg SC |
| M-Site 4 | Thoracotomy involving the dissection of intercostal muscles | ECG, body temperature, SpO_2_, controlled ventilation | 37.0-37.5 °C | | 7-0 silk, 9mm needle, 3/8 circle | | Buprenorphine 0.05 mg/Kg SC |
| M-Site 5 | Thoracotomy involving the dissection of intercostal muscles | ECG, PiP/PEEP, body temperature | 37.0-37.5 °C | | 6-0 silk, 10mm needle | | Buprenorphine 0.075 mg/Kg SC |

Electrocardiogram, ECG; Subcutaneous, SC; peak inspiratory pressure, PiP; positive end expiratory pressure, PEEP.

**Table S4B.** Comparative overview of surgical techniques across rat sites.

|  | **Surgery** | **Parameters monitored during surgery** | **Temperature**  **range** | **Suture type for occlusion** | **Analgesia** |
| --- | --- | --- | --- | --- | --- |
| R-Site 1 | Thoracotomy involving the dissection of intercostal muscles | ECG, body temperature | 36.5-37.5 °C | 5-0 Prolene | Buprenorphine 0.05 mg/Kg SC |
| R-Site 2 | Thoracotomy involving the dissection of intercostal muscles | ECG, body temperature, SpO_2_, controlled ventilation | 37.0-37.5 °C | 6-0 silk | Buprenorphine 0.05 mg/Kg SC |
| R-Site 3 | Thoracotomy involving the dissection of intercostal muscles | ECG, body temperature | 36.8-37.5 °C | 5-0 monofilament polypropylene,13mm needle, 1/2 circle | Buprenorphine 0.05 mg/Kg SC |
| R-Site 4 | Thoracotomy involving the dissection of intercostal muscles | ECG, body temperature, controlled ventilation | 37.2-37.6 °C | 5-0 monofilament polypropylene | Buprenorphine 0.05 mg/Kg SC |

Electrocardiogram, ECG; Subcutaneous, SC.

**Table S5A.** Infarct staining details across mice sites.

|  | **Euthanasia** | **Area-at-risk, procedure** |
| --- | --- | --- |
| M-Site 1 | Pentobarbital, 90 mg/Kg IP | In vivo, delineation of non-ischemic myocardial tissue was achieved by administering EB dye via apical puncture into the left ventricle. |
| M-Site 2 | Isoflurane 5% | In vivo, delineation of non-ischemic myocardium by perfusing EB dye through the aorta. |
| M-Site 3 | Pentobarbital, 100mg/Kg IP | In vivo, delineation of non-ischemic myocardial tissue was achieved by administering EB dye via apical puncture into the left ventricle. |
| M-Site 4 | Ketamine, 100 mg/Kg  Xylazine, 20 mg/Kg | Ex vivo differentiation of non-ischemic myocardial regions by the perfusion of EB dye during aortic cannulation. |
| M-Site 5 | Ketamine, 100 mg/Kg IP  Heparin 1000IU/Kg IP  and cervical dislocation | Ex vivo differentiation of non-ischemic myocardial regions by the perfusion of EB dye during aortic cannulation. |

Intraperitoneal, IP; Evans blue, EB.

**Table S5B.** Infarct staining details across rat sites.

|  | **Euthanasia** | **Area-at-risk, procedure** |
| --- | --- | --- |
| R-Site 1 | Pentobarbital, 60 mg/Kg IP | Ex vivo differentiation of non-ischemic myocardial region using the Langendorff system |
| R-Site 2 | Ketamine, 100 mg/Kg IP  Xylazine, 20 mg/Kg IP | Ex vivo differentiation of non-ischemic myocardial regions by the perfusion of EB dye during aortic cannulation. |
| R-Site 3 | Pentobarbital, 100mg/Kg IP  Heparin 1000 IU/Kg IP | Ex vivo differentiation of non-ischemic myocardial regions by the perfusion of EB dye during aortic cannulation. |
| R-Site 4 | Ketamine, 100 mg/Kg IP  Xylazine, 10 mg/Kg IP | Ex vivo differentiation of non-ischemic myocardial regions by the perfusion of EB dye during aortic cannulation. |

Intraperitoneal, IP; Evans blue, EB.

**Table S6A.** Animal exclusions based on pre-defined criteria

|  | M-Site 1 | M-Site 2 | M-Site 3 | M-Site 4 | M-Site 5 | R-Site 1 | R-Site 2 | R-Site 3 | R-Site 4 |
| --- | --- | --- | --- | --- | --- | --- | --- | --- | --- |
| Sham | 2 | 0 | 0 | 0 | 0 | 1 | 0 | 0 | 1 |
| Control | 2 | 0 | 2 | 2 | 2 | 3 | 1 | 3 | 4 |
| IPC | 8 | 0 | 2 | 0 | 1 | 4 | 0 | 4 | 2 |
| Total | 12 | 0 | 4 | 2 | 3 | 8 | 1 | 7 | 7 |

**Table S6B**. Mortality rates at the mice and rat sites.

| Site | **Control** | |  | **IPC** | |  | **Sham** | | |
| --- | --- | --- | --- | --- | --- | --- | --- | --- | --- |
|  | ***Dead*** | ***Survived*** | ***Total***  ***animals*** | ***Dead*** | ***Survived*** | ***Total animals*** | ***Dead*** | ***Survived*** | ***Total***  ***animals*** |
| M-Site 1 | 2 | 10 | 12 | 8 | 4 | 12 | 2 | 4 | 6 |
| M-Site 2 | 0 | 10 | 10* | 0 | 10 | 10* | 0 | 6 | 6 |
| M-Site 3 | 2 | 10 | 12 | 2 | 10 | 12 | 0 | 6 | 6 |
| M-Site 4 | 0 | 8 | 8 | 0 | 8 | 8 | 0 | 6 | 6 |
| M-Site 5 | 2 | 10 | 12 | 1 | 12 | 13 | 0 | 6 | 6 |
|  |  |  |  |  |  |  |  |  |  |
|  | **Control** | |  | **IPC** | |  |  | ***Sham*** |  |
|  | ***Dead*** | ***Survived*** | ***Total***  ***animals*** | ***Dead*** | ***Survived*** | ***Total animals*** | ***Dead*** | ***Survived*** | ***Total***  ***animals*** |
| R-Site 1 | 3 | 10 | 13 | 3 | 9 | 12 | 1 | 5 | 6 |
| R-Site 2 | 0 | 12 | 12 | 0 | 12 | 12 | 0 | 6 | 6 |
| R-Site 3 | 3 | 9 | 12 | 3 | 8 | 11 | 0 | 6 | 6 |
| R-Site 4 | 4 | 8 | 12 | 2 | 8 | 10 | 1 | 5 | 6 |

*** Note: Mortality at this site was observed prior to the assignment of animals to their respective experimental groups, therefore, it has not been included in this table.

**Table S7A**. Causes of death for each group across the five mouse sites.

| *Site* | ***Causes of death (where known)*** | ***Group*** |
| --- | --- | --- |
| *M-Site 1* | 1x died during chest closure, likely caused by pneumothorax resulting from a chest wall puncture.  1x died after chest closure, during reperfusion, likely caused by pneumothorax resulting from a chest wall puncture. | *Sham* |
|  | 1x died after 24h reperfusion, during re-anaesthesia with pentobarbital, cause: unknown.  1x died after re-anaesthesia with pentobarbital, cause: unknown | *Control* |
|  | 1x died during early reperfusion (15-20 min); cause: unknown.  1x died during ischemic preconditioning; cause unknown.  1x died before Evans blue staining, cause unknown; excluded due to suture failure.  1x died after chest closure during early reperfusion (15-20 min), causing probable pneumothorax due to a hole in the chest.  1x died after ischemic preconditioning at the onset of ischemia; cause: unknown.  1x died during ischemia; cause: unknown.  1x died 4 minutes after ischemia induction; cause unknown.  1x died after chest closure during early reperfusion (15-20 min), causing probable pneumothorax due to a hole in the chest. | *IPC* |
| *M-Site 2* | 4x died during surgery due to anesthesia and surgery failure  1x died due to failed intubation | *Not stated* |
| *M-Site 3* | 1x died during recovery, stopped breathing few hours after surgery  1x died during ischemia, severe bleeding | *Control* |
|  | 1x died during ischemia, cause unknown  1x died during recovery, stopped breathing few hours after surgery | *IPC* |
| *M-Site 4* | No deaths | *Control* |
| *M-Site 5* | 1x died next morning after the surgery  1x died next morning after the surgery | *Control* |
|  | 1x died due to severe bleeding during IPC maneuver | *IPC* |

**Table S7B**. Causes of death for each group across the four rat sites.

| *Site* | ***Causes of death (where known)*** | ***Group*** |
| --- | --- | --- |
| *R-Site 1* | 1x died in recovery period after stop of ventilation | *Sham* |
|  | 1x died during reperfusion  1x died during ischemia | *Control* |
|  | 1x died during early reperfusion  1x died in recovery period before end of ventilation  1x died before ischemia during IPC in AV block | *IPC* |
| *R-Site 2* | No deaths | *Control* |
| *R-Site 3* | 1x died due to stopping breathing a few hours post-surgery.  1x found dead the morning after surgery.  1x died due to bleeding following the surgery. | *Control* |
|  | 3x found dead the morning after surgery. | *IPC* |
| *R-Site 4* | 1x died at 5 min after sham ischemia; cause: non-arrhythmic | *Sham* |
|  | 1x died at 20 min of ischemia, cause: non-arrhythmic  1x died at 5 min of reperfusion; cause: non-arrhythmic  1x died at 20 hours of reperfusion; cause: non-arrhythmic  1x died at 3rd cycle of IPC; cause: non-arrhythmic | *Control* |
|  | 1x died at 5 hours of reperfusion; cause: VF  1x died during the induction of ischemia; cause: bleeding | *IPC* |
